# Cardiac rehabilitation versus standard care after aortic aneurysm repair (Aneurysm CaRe): study protocol for a randomised controlled trial

**DOI:** 10.1186/s13063-015-0669-2

**Published:** 2015-04-15

**Authors:** Sandeep S Bahia, Peter J Holt, Kausik K Ray, Michael Ussher, Jan D Poloniecki, Rajan Sharma, Matthew J Bown, Robert J Hinchliffe, Matthew M Thompson, Alan Karthikesalingam

**Affiliations:** Department of Outcomes Research, St George’s Vascular Institute, St George’s Healthcare NHS Trust, Room 4.007, Blackshaw Road, London, SW17 0QT UK; Population Health Research Institute, St George’s University of London, London, SW17 0RE UK; Department of Cardiovascular Sciences, St George’s University of London, London, SW17 0RE UK; Department of Cardiovascular Sciences and NIHR Leicester Cardiovascular Biomedical Research Unit, University of Leicester, Robert Kilpatrick Clinical Sciences Building, Leicester Royal Infirmary, Leicester, LE2 7LX UK

**Keywords:** Aorta, aneurysm, cardiovascular risk, survival

## Abstract

**Background:**

Abdominal and thoracic aortic aneurysms (A/TAA) are an important cause of mortality amongst the older population. Although A/TAA repair can be performed with low peri-operative risk, overall life expectancy remains poor in the years that follow surgery. The majority of deaths are caused by heart attack or stroke, which can both be prevented by cardiac rehabilitation (CR) in patients with clinically-manifest coronary artery disease. A Cochrane review has urged researchers to widen the use of CR to other populations with severe cardiovascular risk, and patients surviving A/TAA repair appear ideal candidates. However, it is unknown whether CR is feasible or acceptable to A/TAA patients, who are a decade older than those currently enrolling in CR. Aneurysm-CaRe is a feasibility randomised controlled trial (RCT) that will address these issues.

**Methods and design:**

Aneurysm-CaRe is a pilot RCT of CR versus standard care after A/TAA repair, with the primary objectives of estimating enrolment to a trial of CR after A/TAA repair and estimating compliance with CR amongst patients with A/TAA. Aneurysm-CaRe will randomise 84 patients at two sites. Patients discharged from hospital after elective A/TAA repair will be randomised to standard care or enrolment in their local CR programme with a protocolised approach to medical cardiovascular risk reduction. The primary outcome measures are enrolment in the RCT and compliance with CR. Secondary outcomes will include phenotypic markers of cardiovascular risk and smoking cessation, alongside disease-specific and generic quality-of-life measures.

**Trial registration:**

ISRCTN 65746249 5 June 2014

## Background

Abdominal and thoracic aortic aneurysms (A/TAAs) are a significant health problem that account for approximately 4 deaths per 100,000 population per year [1]. A/TAA repair can be performed with low peri-operative risk [2] and is an effective means of mitigating the long-term risk of mortality caused by A/TAA rupture. However, overall life expectancy remains poor in the years that follow surgery because patients with A/TAA have widespread atherosclerosis and considerable cardiovascular risk factors, which cause an unusually high rate of heart attacks, strokes and major amputation [3] compared with the wider population [4,5].

Patients with A/TAA have similar cardiovascular risk factors to those with clinically-manifest coronary artery disease (CAD) and might benefit from the application of similar therapeutic strategies for secondary prevention. High-quality evidence has demonstrated that cardiac rehabilitation (CR) improves outcomes after cardiac surgery or myocardial infarction, improving adherence to evidence-based care and both the quality and quantity of life [6]. CR is a complex intervention delivered typically over 8–12 weeks, which combines supervised exercise, dietary and lifestyle modification, smoking cessation, and optimal medical therapy for cardiovascular risk factors. This programme is very cost-effective: CR is the third most economic intervention for reducing cardiovascular mortality, after aspirin and beta-blockers [7]. Extensive national infrastructure is in place for delivering this service, and a Cochrane review has urged researchers to apply CR in other populations with severe cardiovascular disease [6].

Patients undergoing A/TAA repair appear ideal candidates for postoperative CR and are at even greater risk of cardiovascular mortality than patients undergoing cardiac surgery: just 65% remain alive at 5 years after aneurysm repair [8] compared with 90% at 5 years after cardiac surgery [9]. The definitive impact of CR on survival after A/TAA repair could be studied by a multicentre randomised trial of CR after A/TAA repair. This would require 1140 patients to demonstrate an absolute improvement of 9% in 3-year survival, with 90% power, a calculation aiming to bridge the gap in survival between A/TAA patients and age-/gender-matched controls without A/TAA [4]. However, prior to such a considerable undertaking, data are required to estimate the feasibility and efficacy of CR in A/TAA patients.

Aneurysm-CaRe is a pilot randomised controlled trial of CR after A/TAA repair that will address these issues. Patients undergoing A/TAA repair are typically 10 years older than those currently recruited to CR [4,6] and it is therefore not known whether CR would be feasible or acceptable in this group. It also remains unknown whether an intensive programme of CR can improve phenotypic markers of cardiovascular risk after A/TAA repair. The life expectancy of patients with A/TAA may not be modifiable by CR and the equipoise justifies a trial such as Aneurysm-CaRe. Cigarette smoking is strongly associated with the development of A/TAA and is the most important modifiable risk factor for patients with vascular disease [10]. The period after A/TAA surgery presents a unique window for preventing a return to smoking [11] and enrolment in a CR programme provides a novel opportunity for quantifying the effects of exercise on smoking cessation in patients with A/TAA [12]. Standard care may provide as few as 40% of A/TAA patients with appropriate cardiovascular risk management (using antiplatelet agents, statins, angiotensin-converting enzyme inhibitors or beta-blockers) [13] and it is likely that attainment of risk-factor targets is even lower than in comparable CAD populations. These data highlight an unmet clinical need. Aneurysm-CaRe presents an important opportunity to improve life expectancy in a high-risk population (A/TAA patients) using an existing national service (CR) and the accompanying infrastructure.

## Methods

### Trial design and objectives

Aneurysm-CaRe is a pilot randomised controlled trial comparing CR with standard care after elective A/TAA repair. It is envisaged that this study will inform Aneurysm-CaRe II: a large-scale definitive phase III randomised controlled trial of CR after elective A/TAA repair.

The objectives of Aneurysm-CaRe are to quantify the proportion of patients surviving A/TAA repair that agree to enrol in a RCT of CR and to quantify compliance with CR in terms of attendance amongst those randomised to CR. The secondary objectives of Aneurysm-CaRe are to quantify the effect of CR compared with standard care on phenotypic markers of cardiovascular risk, patient behaviour/biometrics and quality of life. Finally, Aneurysm-CaRe aims to enable optimisation of the patient pathway for a definitive RCT and identification of pitfalls such as exposure bias or methodological heterogeneity.

### Ethical approval

Full ethical approval has been gained from the NRES Committee London (Bloomsbury), reference 13/LO/0395.

### Funding

Aneurysm-CaRe is supported by a British Heart Foundation Project Grant (PG/13/98/30490).

### Randomisation

**Figure 1:**
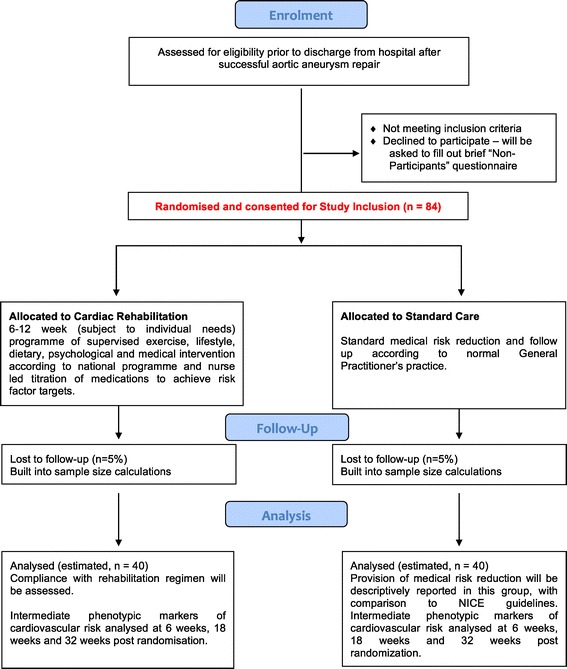
CONSORT 2010 flow diagram for pilot randomised controlled trial of cardiac rehabilitation vs. standard care after aortic aneurysm repair (Aneurysm-CaRe).

Patients discharged from hospital after elective endovascular or open thoracic or abdominal aortic aneurysm repair will be randomised, in a 1:1 ratio, between the intervention arm (“Cardiac Rehabilitation – CR”) and the control arm (“Standard Care”) (Figure 1). Secure Internet-based central randomisation will be provided by an NIHR-accredited clinical trials unit. A minimisation algorithm will be utilised to ensure stratification variables (age, gender and study site) are balanced between groups. Each site and randomiser will be provided with a unique login and password to allow access to the randomisation service.

### Inclusion criteria

All patients who are discharged home alive after undergoing elective thoracic or abdominal aortic aneurysm repair at St George’s Vascular Institute or Leicester Royal Infirmary during the recruitment period will be eligible for participation and offered the chance to enrol.

### Exclusion criteria

The exclusion criteria are:

1. Any patient under the age of 50 years, or with aortic pathology secondary to connective tissue disease (including Marfan’s and Loeys-Dietz syndromes)
2. Anyone unable or unwilling to consent to participation
3. Anyone unable or unwilling to attend CR sessions
4. Any patient deemed too unfit for the CR programme by the Lead CR nurse
5. Patients undergoing expedited or emergent surgical intervention for symptomatic or ruptured aortic aneurysms

A pseudonymised registry will be kept to record the proportion of patients undergoing elective aneurysm repair but meeting exclusion criteria for Aneurysm-CaRe during the study period.

### Primary outcome measures

1. Enrolment: the proportion of patients surviving A/TAA repair and meeting Aneurysm CaRe inclusion criteria, that agree to enrolment in the feasibility RCT.
2. Compliance with CR therapy in the group randomised to CR (defined as the proportion of patients demonstrating attendance at 100% of scheduled CR sessions over 8–12 weeks; the period of CR offered is dependent on individual patient needs).

### Secondary outcome measures

1. Time to death.
2. Time to first adverse cardiovascular event after aneurysm repair.
  The composite “adverse cardiovascular event” outcome includes: non-fatal myocardial infarction, stroke, non-traumatic amputation and revascularisation. The revascularisation endpoint comprises strictly defined coronary procedures (coronary artery bypass graft surgery, percutaneous coronary interventions and percutaneous coronary interventions) and peripheral vascular procedures (embolectomy, bypass and angioplasty). In the absence of relevant COMET outcomes (www.comet-initiative.org), this outcome measure mirrors the composite cardiovascular endpoint measured for previous randomised RCTs of cardiovascular risk reduction [14]. Analysis of the “composite cardiovascular disease event” endpoint will be adjusted to account for “competing risks”, for example, the intervention reducing the risk of one event but increasing the risk of another [15]. Outcomes will be compared with the BHF-funded National Audit of CR for attendance, adherence, and outcomes with MI, PCI and CABG patients.
3. Intermediate phenotypic markers of cardiovascular risk measured at enrolment, 12-weeks after randomisation to CR and 6-months after randomisation to CR or standard care: serum lipids (total cholesterol, HDL and LDL), systolic and diastolic blood pressure, HbA1C, BNP, troponin, hsCRP, fibrinogen, homocysteine, urate, eGFR, serum insulin levels and echocardiographic measures of cardiac function. We will also be testing for urinary microalbuminuria at each time point.
4. Indicators of patient behaviour and lifestyle, and patient biometrics, including: waist:hip ratio, waist circumference, BMI, ankle-brachial pressure index (ABPI) and toe pressures. Smoking cessation outcomes will include: salivary cotinine, expiratory carbon monoxide (<10 ppm), “prolonged abstinence” [16] and attendance at NHS stop-smoking services. Physical activity levels will be assessed by a 7-day-recall interview [17].
5. Changes in patients’ attitudes to cardiovascular risk and their knowledge of their condition.
6. Quality of life: EQ5D5L questionnaires will be administered at baseline, enrolment, 12-weeks after randomisation to CR or standard care and 6 months after randomisation to CR or standard care.
7. Cost-effectiveness: Mean costs and quality adjusted life years will be compared on an intention-to-treat basis. The perspective will be the NHS and PSS. The analysis will include hospital re-admissions and pharmaceuticals. Unit cost will be obtained from the published literature.

### Power calculation

A pilot RCT of 84 patients is planned; to estimate enrolment rates in the trial, we plan to approach 140 patients for consent. We expect that at least 60% (95% confidence interval 50.7%-68.8%) will agree to enrolment, leading to the randomised study of 84 patients. The lower margin of the 95% confidence interval for this enrolment rate (50.7%) provides adequate precision to establish that at least 50% of aortic aneurysm patients will agree to participate in an RCT of CR; establishing the feasibility of a definitive multi-centre national RCT. The study will cease recruitment when either 84 patients have been randomised or 140 patients approached. The feasibility trial is itself feasible because St George’s and Leicester perform in excess of 200 thoracic or abdominal aneurysm repairs per annum. The target enrolment is realistic because previous RCTs of A/TAA surgery in the UK have reported 74-76% enrolment rates (EVAR-1 and EVAR-2; NIHR HTA trials [18,19]).

### Patient pathway

**Figure 2:**
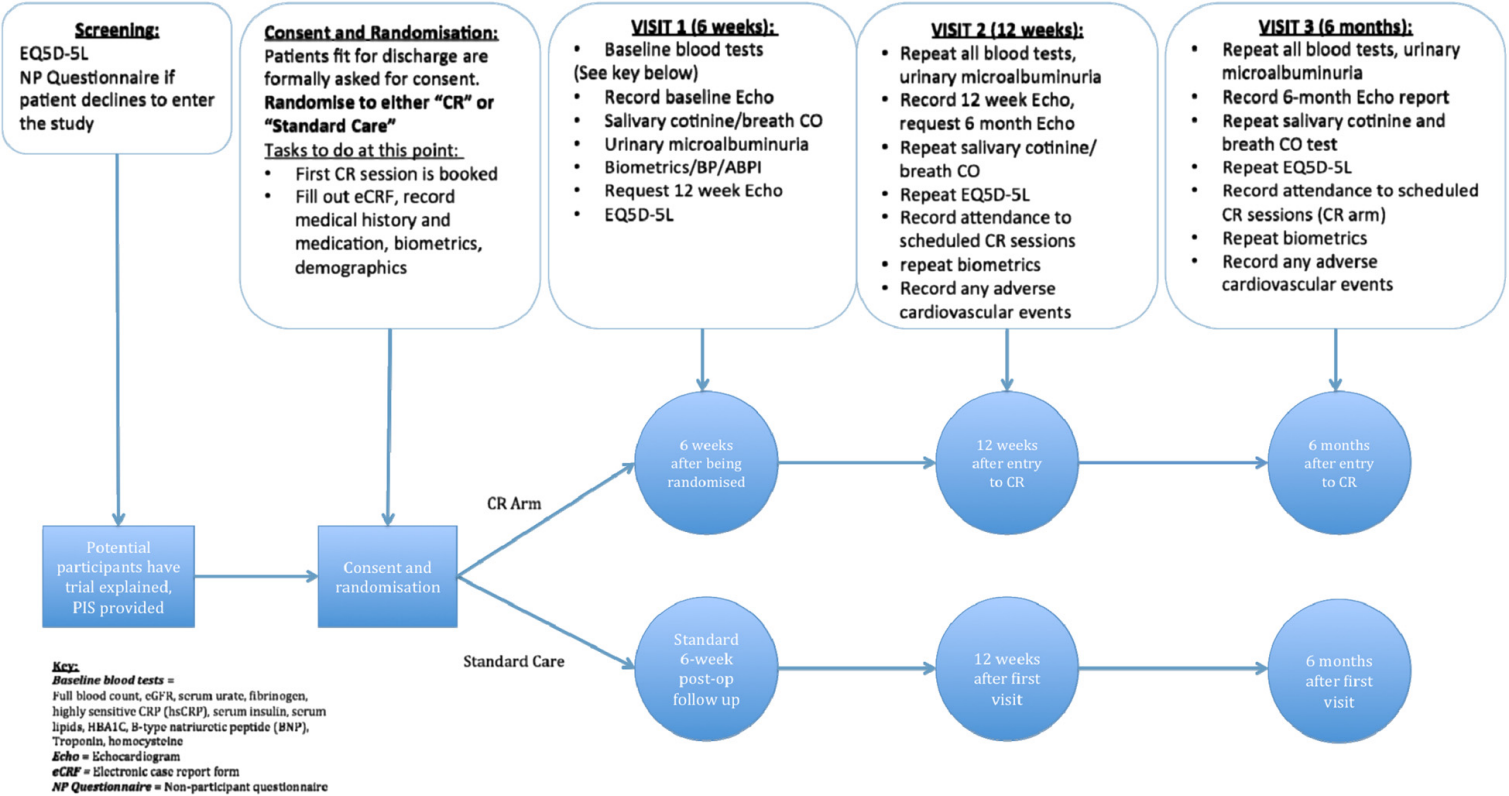
Patient pathway.

**Figure 3:**
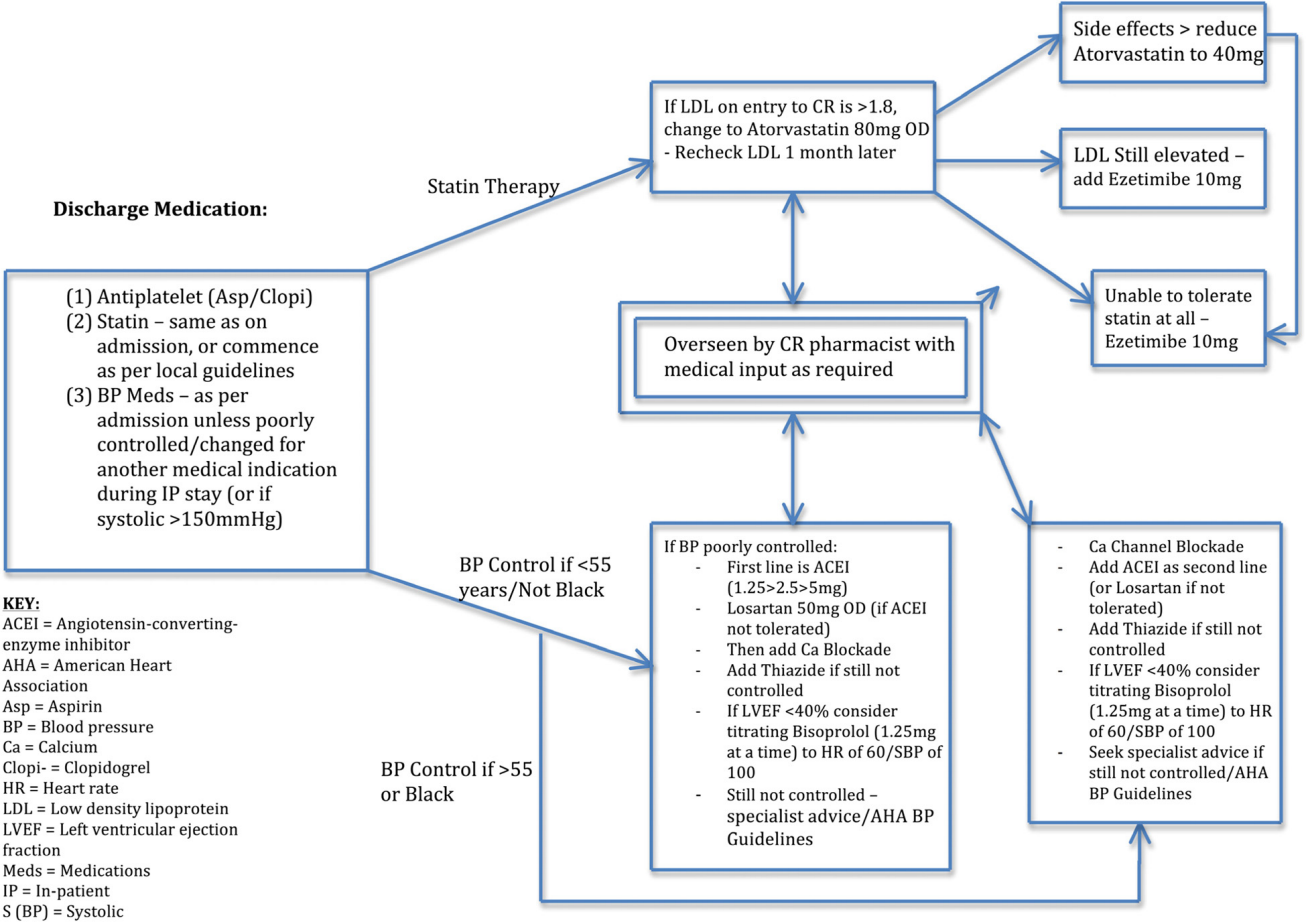
Protocol for medical management of cardiovascular risk factors in patients randomised to CR group.

The pathway is outlined in Figures 1, 2 and 3.

### Informed consent

Patients will be identified and given information about the trial from three settings and seen by the local Principal Investigator, a Research Investigator or the Lead CR Nurse:

1. At the surgical outpatient clinic when it is certain that aortic aneurysm repair will be scheduled. The trial will be discussed with the patient and the patient given a Patient Information Sheet.
2. At the pre-operative assessment clinic.
3. On admission for surgery, prior to aortic aneurysm repair.

Formal written consent will be obtained after the patient has undergone aortic aneurysm repair and been deemed fit for discharge from hospital.

### Randomisation

After informed consent has been obtained, a nominated research investigator will randomise the patient using a secure online NIHR-accredited Clinical Trials Unit Randomisation Service (King’s Clinical Trials Unit). The patient will be allocated a unique trial identification PIN number. Neither investigators nor patients will be blinded to treatment allocation. The patient’s GP will be notified of the treatment allocation, and supplied with a reminder letter highlighting current NICE guidelines for the medical management of cardiovascular risk in patients with peripheral vascular disease.

### Treatment and follow-up

Patients randomised to CR will be seen by a member of the Cardiac Rehabilitation team on the ward prior to discharge if possible, or at the induction CR meeting scheduled 4–6 weeks after aortic aneurysm repair. The CR Arm will receive a protocolised approach to intensive medical risk factor reduction (Figure 3) in addition to enrolment in the local multimodal Cardiac Rehabilitation programme, which should follow national guidelines (www.cardiacrehabilitation.org.uk). CR is an existing national service, comprising a protocolised 8–12 week regime of supervised exercise, lifestyle modification, dietary interventions, psychological assessment and medical risk modification. Each weekly session takes 1 to 2 h and is supervised by two nurses trained in CR, two qualified physiotherapists and one occupational therapy technician. Patients assessed as being low-risk attend sessions with a maximum of 20 patients; those deemed high-risk attend sessions with 12 patients. Each session begins with a “check-in” consisting of a blood pressure and heart rate check and a review of each patient’s progress to date. The group then proceed through a supervised warm-up of 10–15 min, followed by the main exercise session of 45 min. This is followed by a warm-down, and finally a “check-out” consisting of further blood pressure and heart rate measurement.

An individual assessment is made of each patient’s progress and needs; in some cases this involves extending the duration of the patient’s participation in the programme beyond the initial 8 weeks. The patients that have been randomised to “treatment as usual” will receive “standard” follow-up, i.e. routine post-operative out-patient appointments and/or follow-up scans as necessary and GP-based management of their co-existing co-morbidities such as hypertension. Local standards for routine outpatient care after A/TAA repair will be unaffected by the trial, including existing appointments for endograft surveillance and vascular surgery outpatient review.

Patients in both arms of the study will undergo blood tests, urinalysis, salivary testing, biometric assessment and quality-of-life assessment at 6 weeks after surgery (entry to CR in the CR group) (Figure 2). These tests will be repeated, in addition to follow-up echocardiography, in all patients at 12 weeks after randomisation to CR (or 12 weeks after surgery for those randomised to standard care) and 6 months after randomisation into CR (or 6 months after surgery for “standard care” patients).

- Blood tests will comprise hsCRP, troponin, BNP, lipid profile, fibrinogen, urate, eGFR, HBA1C and serum insulin.
- Patients who smoke will have salivary cotinine testing conducted and breath carbon monoxide testing on entry to CR (or 6 weeks after randomisation in the standard care group), 12-weeks after randomisation to CR and at 6-month follow-up.

## Discussion

Given that this study straddles specialties, particularly cardiology and vascular surgery, an extensive collaborative approach has been employed to the benefit of the study design and patient pathway. Pragmatically, rather than trying to standardise the cardiac rehabilitation (CR) received by patients not only at the two sites, but also at individual CR locations within those sites, we have adopted the view that the existing programmes at each venue will be followed. National CR guidelines are already in place.

An electronic Case Report Form (CRF) has been developed in conjunction with the Kings College Clinical Trials (KCTU) unit to ensure consistency in data inputting and to streamline the analysis of results. This will also allow assessment of the practicalities of using such an electronic database across multiple sites. Randomisation will be carried out electronically using an online randomisation tool developed by KCTU that is accessible at both sites. A Trial Manager has been appointed to oversee the study, based at St George’s.

## Trial status

This trial commenced recruiting patients in September 2014 at both sites.

## Abbreviations

A/TAA: Abdominal aortic aneurysm
ABPI: Ankle-brachial pressure index
BMI: Body mass index
BNP: Brain natriuretic protein
CR: Cardiac rehabilitation
CRF: Case report form
eGFR: Estimated glomerular filtration rate
EVAR: Endovascular aneurysm repair
EQ5D5L: EuroQol questionnaire
GP: General practitioner
HBA1C: Glycosylated haemoglobin
HTA: Health technology assessment
hsCRP: Highly sensitive C-reactive protein
KCTU: King’s clinical trials unit
NICE: National Institute for Health and Care Excellence
NIHR: National Institute for Health Research
TAA: Thoracic aortic aneurysm
